# Comprehensive analysis of *NuMA *variation in breast cancer

**DOI:** 10.1186/1471-2407-8-71

**Published:** 2008-03-10

**Authors:** Outi Kilpivaara, Matias Rantanen, Anitta Tamminen, Kristiina Aittomäki, Carl Blomqvist, Heli Nevanlinna

**Affiliations:** 1 Department of Obstetrics and Gynecology, Helsinki University Central Hospital (HUCH), Helsinki, Finland; 2 Department of Clinical Genetics, Helsinki University Central Hospital (HUCH), Helsinki, Finland; 3 Department of Oncology, Helsinki University Central Hospital (HUCH), Helsinki, Finland; 4 Dept. Oncology, Uppsala University Hospital, Sweden

## Abstract

**Background:**

A recent genome wide case-control association study identified *NuMA *region on 11q13 as a candidate locus for breast cancer susceptibility. Specifically, the variant Ala794Gly was suggested to be associated with increased risk of breast cancer.

**Methods:**

In order to evaluate the *NuMa *gene for breast cancer susceptibility, we have here screened the entire coding region and exon-intron boundaries of *NuMa *in 92 familial breast cancer patients and constructed haplotypes of the identified variants. Five missense variants were further screened in 341 breast cancer cases with a positive family history and 368 controls. We examined the frequency of Ala794Gly in an extensive series of familial (n = 910) and unselected (n = 884) breast cancer cases and controls (n = 906), with a high power to detect the suggested breast cancer risk. We also tested if the variant is associated with histopathologic features of breast tumors.

**Results:**

Screening of *NuMA *resulted in identification of 11 exonic variants and 12 variants in introns or untranslated regions. Five missense variants that were further screened in breast cancer cases with a positive family history and controls, were each carried on a unique haplotype. None of the variants, or the haplotypes represented by them, was associated with breast cancer risk although due to low power in this analysis, very low risk alleles may go unrecognized. The *NuMA *Ala794Gly showed no difference in frequency in the unselected breast cancer case series or familial case series compared to control cases. Furthermore, Ala794Gly did not show any significant association with histopathologic characteristics of the tumors, though Ala794Gly was slightly more frequent among unselected cases with lymph node involvement.

**Conclusion:**

Our results do not support the role of *NuMA *variants as breast cancer susceptibility alleles.

## Background

Recently, a genome-wide association study with over 25 000 single-nucleotide polymorphisms (SNP) was conducted to discover variants associated with increased breast cancer risk [1]. The initial sample set comprised of 254 German breast cancer cases and 268 controls [1]. Fifty-two SNPs were selected for replication genotyping in two independent sample series, one German (188 cases, 150 controls) and one Australian (180 cases, 180 controls) and, among others; a putative breast cancer susceptibility locus was identified at 11q13. The variant with strongest association in the 300 kb region was SNP (rs673478) in an intron of gene *LOC220074*, *p *= 0.011 and OR = 1.59 (for combined discovery and replication sets). The high-linkage disequilibrium 300 kb-block was fine-mapped using an additional set of SNPs. The block contains seven annotated genes of which the *NuMA *gene spanning ~75 kb in the center of the block was chosen as a candidate for breast cancer association. Four SNPs from the fine-mapping SNP set in *NuMA *were carefully chosen and genotyped in all three sample sets. The strongest association with breast cancer was observed for SNP rs3750913 located in exon 16, which leads to an amino acid substitution Ala794Gly in NuMA protein (*p *= 0.002, OR = 2.13, for combined sample sets). Furthermore, Kammerer at al. reported an even stronger association for Ala794Gly with familial breast cancer: comparison of frequency in cases with family history of cancer with controls is reported to result in *p*-value of 0.001 and OR = 4.45 [1].

*NuMA *(*Nu*clear *M*itotic *A*pparatus protein) gene is located on chromosome 11q13 and it encodes a 236 kDa nuclear protein essential for normal mitotic spindle organization. NuMA protein consists of globular head and tail domains and a separating long coiled-coil domain [2] which mediates dimerization and oligomerization of NuMA [3]. The tail domain itself is bipartite including a region critical for interaction with the mitotic spindle and another region needed for accurate nuclear reformation including a nuclear localization signal [4-6]. Expression of NuMA with deletion in either head or tail domains results in dominant defects in mitosis [7].

*NuMA *has been associated with acute promyelocytic leukemia (APL). In very rare cases of APL translocation NuMA-RARα (retinoic acid receptor α) t(11;17)(q13;q21) has been observed instead of the common translocation involving a gene fusion of promyelocytic leukemia protein (PML) and RARα [8]. The fusion protein consists of 1883 amino acids of NuMA protein in the aminoterminal end the rest of the total 2285 amino acid protein being derived from RARα – the structure similar to all RARα fusion proteins seen in APL. The essential feature of the fusion partner NuMA is to be capable of establishing protein-protein interactions that may result in formation of defective heterodimers interfering with retinoid signaling [8]. It has been reported that NuMA is an interaction partner of GAS41 (glioma-amplified sequence 41) [9], which is a highly conserved protein and a putative transcription factor amplified in even at early stages of human glioma [10]. This interaction links the structural protein NuMA to the regulation of gene expression. In addition to the previously mentioned studies the role of NuMA has not been under extensive research in association with cancer.

In this study, we screened the *NuMA *gene for genetic variants in breast cancer cases and studied their relevance for breast cancer risk. In particular, we studied the association of the reported breast cancer susceptibility allele NuMA Ala794Gly in larger series of unselected and familial breast cancer cases and controls, and examined the association of this variant with clinicopathologic characteristics of breast tumors.

## Methods

### Breast cancer patients

The 28 exons and exon-intron boundaries as well as 5' and 3' untranslated regions (ENST00000358965) were screened for sequence variants in a total of 92 familial (non-BRCA1/2) breast cancer cases. Familial breast cancer case was here, in this initial screening, defined as three or more breast cancer cases in first degree family members (including the index case).

A number of identified variants were selected, based on bioinformatic analyses, for further exploratory screening in familial breast cancer patients (n = 341) and healthy population controls (n = 368). The previously identified possible risk variant Ala794Gly was screened in larger series of familial (n = 910) and unselected breast cancer cases (n = 884), and compared in frequency with population controls (n = 906). The unselected breast cancer case series included consecutive newly diagnosed breast cancer patient samples collected at the Department of Oncology, Helsinki University Central Hospital at two separate periods in 1997–1998 and 2000 covering 79% of all newly diagnosed breast cancer cases at respective study periods combined (for more detailed description, see [11,12]. The series of familial breast cancer cases in this study have been collected at the Helsinki University Central Hospital as described in [13]. The successfully genotyped series included 378 patients with strong family history, defined as three or more breast or ovarian cancer cases in the first or second degree family members including the index case. The latter series were screened negative for BRCA1/2 mutations (previously described in detail in [14-16]. The remaining 515 successfully genotyped familial cases had a single affected first degree family member; for 312 of these cases, the Finnish BRCA1/2 founder mutations have been excluded as described [14,15]. The cancer diagnoses have been verified through the Finnish Cancer Registry and hospital records.

Histopathologic data were collected from pathology reports for all the primary breast tumors available from the unselected case series (889 tumors from 842 successfully genotyped cases). The data set in this study includes information on tumor histology, grade, estrogen and progesterone receptor status, p53 immunohistochemistry, tumor diameter, lymph node status, distant metastases, and the age at the time of diagnosis.

The study was performed with informed consent from the patients and permissions from the Ethics Committee of the Department Obstetrics and Gynecology, as well as from the Ministry of Social Affairs and Health in Finland.

### Variant screening

All NuMA exons were screened for sequence variants using single strand conformation polymorphism (SSCP) or conformation sensitive gel electrophoresis (CSGE) method. For further screenings restriction fragment polymorphism (RFLP) method was used when applicable. Variant Arg972Gln (R972Q) was screened by RFLP using enzyme BsrBI, Arg1471Trp (R1471W) using enzyme MspI), and Arg1665Cys (R1665C) using enzyme HhaI. For the screening of intronic variant IVS2+34G > C a mutagenesis primer was designed to create a restriction site for enzyme NdeI. Primer sequences are available on request. All endonucleases are products of New England BioLabs, Beverly, MA. All variants found in the screening have been confirmed by either DNA sequencing or repeating the screening by respective method (SSCP, CSGE, RFLP) for the genomic DNA sample. The genotyping Ala794Gly has been performed using Amplifluor™ fluorescent genotyping (K-Biosciences, Cambridge, UK). The quality of the Ala794Gly genotyping was ascertained by analyzing duplicate samples (92 samples were genotyped with 100% concordance). Genotyping success rate for unselected cases was 95% (842/884), for familial cases 98% (893/910), and 100% for population controls (906/906)]. The unsuccesfull genotypes were due to poor quality or lack of DNA in the analysis.

### Statistical and Bioinformatic Methods

Standard chi squared or Fisher exact tests were used to assess the differences in genotype frequencies between groups. Per allele odds ratios for each SNP, together with 95% confidence intervals, were estimated using logistic regression. Differences in survival by genotype were assessed using the log-rank test. Analyses were performed using SPSS (SPSS Inc., Chicago, IL). All *p*-values are two-sided. SIFT [17,18] and PolyPhen [19,20] were used for predicting the impact of observed amino acid substitutions on the structure and function of NuMA protein. The most probable haplotypes were reconstructed using the program PHASE [21-23].

## Results

A total of 11 exonic and 12 intronic or untranslated region (UTR) variants in NuMA were identified (Table 1). We selected 5 of these variants, in addition to Ala794Gly, for further analysis. These five variants had a SIFT score lower than or similar to the score for Ala794Gly (SIFT score 0.29). In particular, two variants (Arg218Trp and Arg1471Trp) had a SIFT score was 0.00, which may indicate that the amino acid substitution is harmful to the protein. We genotyped these variants in 341 familial breast cancer patients and 368 population controls. All were present at a similar frequency in cases and controls. (Table 2).

**Table 1:** Variants identified in NuMA and their predicted effect on protein level by SIFT and PolyPhen

**Location**	**Variant**	**Frequency**	**SIFT**	**PolyPhen**
IVS2	IVS2+34 G > C	4/92		
IVS4	IVS4+46 delCA	19/92		
IVS5	IVS5+23 T > A	3/92		
IVS5	IVS5-18 G > A	19/92		
IVS6	IVS6-53 A > G	19/92		
IVS8	IVS8-52 T > A	19/92		
ex 10	652 C > T, Arg218Trp	1/92	0.00	unknown
IVS12	IVS12-37 insA	19/92		
IVS14	IVS14+32 G > T	9/92		
ex 15	2381 C > G, Ala794Gly	9/92	0.29	benign
ex 15	2484C > T, Gly828Gly	1/92	-	
ex 15	2915 G > A, Arg972Gln	3/92	0.30	benign
ex 15	4012 G > A, Glu1338Lys	1/92	0.28	benign
ex 15	4411 C > T, Arg1471Trp	2/92	0.00	possibly damaging
ex 17	4785 G > A, Lys1595Lys	1/92	-	
ex 18	4996 C > T, Arg1665Cys	1/92	0.03	possibly damaging
IVS18	IVS18+15 C > G	1/92		
ex 20	5335 G > A, Asp1779Asn	3/92	1.00	benign
ex 21	5619 C > T, Ala1873Ala	9/92	-	
ex 25	6083 G > A, Arg2028Gln	1/92	0.94	benign
3' UTR	6517 A > C	10/92		
3' UTR	6519 C > G	10/92		
3' UTR	6580 T > C	1/92		

**Table 2:** Further screening of selected variants in familial breast cancer cases and controls.

**Exon**	**Variant**	**Familial Cases**	**Controls**
10	ex10 652 C > T, Arg218Trp	1/341	1/368
15	ex15 2915 G > A, Arg972Gln	6/333	12/364
15	ex15 4012 G > A, Glu1338Lys	1/323	1/364
15	ex15 4411 C > T, Arg1471Trp	2/339	0/368
18	ex 18 4996 C > T, Arg1665Cys	7/337	6/366

The Ala794Gly variant was present in 5.8% of population controls (53/906), 5.7% of unselected breast cancer cases (48/842) and 4.8% of familial breast cancer cases (43/893) (Table 3). Thus there was no evidence for an association between Ala794Gly genotype and breast cancer risk.

**Table 3:** Results from *NuMA *A794G screening

	**total**	**C:C**	**C:G**	***p***^2^	**OR**^2^	**95%CI**
**Population controls**	906	853(94.2%)	53(5.8%)			
**Unselected cases**	842	794(94.3%)	48(5.7%)	0.89	0.97	0.65–1.45
bilateral breast cancer	54	50(92.6%)	4(7.4%)	0.541^3^	1.35	0.47–3.92
unilateral breast cancer	788	744(94.4%)	44(5.6%)			
multiple cancers^1^	84	81(96.4%)	3(3.6%)	0.617^3^	0.59	0.18–1.93
no multiple cancers	758	713(94.1%)	45(5.9%)			
**Familial cases**	893	850(95.2%)	43(4.8%)	0.33	0.81	0.54–1.23
3+ families	378	359(95.0%)	19(5.0%)	0.56	0.85	0.50–1.46
breast cancer only	298	283(95.0%)	15(5.0%)			
breast and ovarian cancer	80	76(95.0%)	4(5.0%)			
Small families	515	491(95.3%)	24(4.7%)	0.34	0.79	0.48–1.29

^1^case with at least one other cancer than breast cancer

^2^compared to population controls, except comparisons between unilateral vs. bilateral and multiple cancers vs. one cancer

^3^Fisher's exact test

No G:G genotypes were observed.

*NuMA *Ala794Gly did not show any significant association with histopathologic parameters of the tumors (Table 4). There was some evidence of an association between the Ala794Gly variant and positive lymph node status in unselected breast cancer case series (*p *= 0.008), but this association was not seen among familial patient series (data not shown). Furthermore, if Bonferroni correction for multiple testing was applied, only *p*-value of 0.006 or smaller would be considered significant. There was no difference in either overall or disease-free survival by Ala794Gly genotype (data not shown).

**Table 4:** Tumor characteristics among 842 unselected breast cancer cases analyzed for *NuMA *A794G.

		***NuMA *A794G (2381C > G)**		*p*
	***Total***	***CC***	***CG***	
Total	n = 889	837(94.2%)	52(5.8%)	
				
Histology	n = 889			ND
Ductal		599(71.6%)	39(75.0%)	
Lobular		132(15.8%)	8(15.4%)	
Medullary		11(1.3%)	1(1.9%)	
other		95(11.4%)	4(7.7%)	
				
Gradus	n = 793			0.107
1		208(27.9%)	7(14.9%)	
2		316(42.4%)	26(55.3%)	
3		222(29.8%)	14(29.8%)	
				
pT Stage	n = 801			0.767 (Fisher)
pT1-pT2		699(92.6%)	44(95.7%)	
pT3-pT4		56(7.4%)	2(4.3%)	
				
Lymph Nodes	n = 858			0.008
pN0		446(55.3%)	360(44.7%)	
pN1-2		19(36.5%)	33(63.4%)	
				
Estrogen Receptor Status	n = 845			0.691
Positive		622(78.4%)	42(80.8%)	
Negative		171(21.6%)	10(19.2%)	
				
Progesterone Receptor Status	n = 846			0.425
Positive		538(67.8%)	38(73.1%)	
Negative		256(32.2%)	14(26.9%)	
				
p53 Status	n = 653			0.035
Positive		132(21.3%)	2(6.1%)	
Negative		488(78.7%)	31(93.9%)	
				
Distant metastasis	n = 853			0.161 (Fisher)
Positive		41(5.1%)	0(0.0%)	
Negative		765(94.9%)	47(100.%)	

The mean age of diagnosis was 57 years for C:C, and 56 for C:G genotypes.

Observed variants in *NuMA *were used for reconstructing haplotypes using PHASE program (Table 5). Altogether 15 different haplotypes defined by 23 sequence variants in *NuMA *were observed in the screening of 92 familial breast cancer patients (Table 5). The most common haplotype (without any observed DNA sequence variants) accounts for 80% of observed haplotypes, and together the three most common haplotypes account for 90% of haplotypes. Each of the missense variants that were further studied represent a unique haplotype except the Ala794Gly which is present in two haplotypes (numbers 12 and 14 in Table 5) defined by an intronic variant IVS2+34 G > C. In order to study the possibility that either of haplotypes 12 or 14 would associate with breast cancer risk we screened the haplotype-defining intronic variant IVS2+34G > C in 337 familial breast cancer cases and 359 controls. Variant IVS2+34G > C was present in cases and controls in similar proportions (cases 49/337, 14.5% and controls 57/359, 16.0%; *p *= 0.6). Furthermore, we did not observe any haplotype 12 carriers in this further screening (variant IVS2+34G > C was always present in Ala794Gly carriers).

**Table 5:** *NuMA *sequence variants and haplotypes in 92 familial breast cancer cases.

Variant																									
**Obs.**	**Freq.**	**Hapl.**	**1**	**2**	**3**	**4**	**5**	**6**	**7**	**8**	**9**	**10**	**11**	**12**	**13**	**14**	**15**	**16**	**17**	**18**	**19**	**20**	**21**	**22**	**23**
148	0.804	1	G	CA	T	G	A	T	C	0	G	C	C	G	G	C	C	G	C	G	C	G	A	C	T
1	0.005	2	G	CA	T	G	A	T	C	0	G	C	C	G	G	C	C	G	C	G	C	G	A	C	**C**
1	0.005	3	G	CA	T	G	A	T	C	0	G	C	C	G	G	C	C	G	C	G	C	**A**	A	C	T
3	0.016	4	G	CA	T	G	A	T	C	0	G	C	C	G	G	C	C	G	C	**A**	C	G	A	C	T
1	0.005	5	G	CA	T	G	A	T	C	0	G	C	C	G	G	C	C	**A**	C	G	C	G	A	C	T
1	0.005	6	G	CA	T	G	A	T	C	0	G	C	C	G	G	C	**G**	G	C	G	C	G	A	C	T
2	0.011	7	G	CA	T	G	A	T	C	0	G	C	C	G	G	**T**	C	G	C	G	C	G	A	C	T
1	0.005	8	G	CA	T	G	A	T	C	0	G	C	C	G	**A**	C	C	G	**T**	G	C	G	A	C	T
3	0.016	9	G	CA	T	G	A	T	C	0	G	C	C	**A**	G	C	C	G	C	G	C	G	A	C	T
1	0.005	10	G	CA	T	G	A	T	T	0	G	C	C	G	G	C	C	G	C	G	C	G	A	C	T
3	0.016	11	G	CA	**A**	G	A	T	C	0	G	C	C	G	G	C	C	G	C	G	C	G	A	C	T
1	0.005	12	G	**delCA**	T	**A**	**G**	**A**	**C**	**insA**	G	C	**T**	G	G	C	C	G	C	G	C	G	**C**	**G**	T
7	0.038	13	G	**delCA**	T	**A**	**G**	**A**	**C**	**insA**	G	**G**	C	G	G	C	C	G	C	G	**T**	G	A	C	T
7	0.038	14	G	**delCA**	T	**A**	**G**	**A**	**C**	**insA**	**T**	C	C	G	G	C	C	G	C	G	C	G	**C**	**G**	T
2	0.011	15	**C**	**delCA**	T	**A**	**G**	**A**	**C**	**insA**	G	**G**	C	G	G	C	C	G	C	G	**T**	G	A	C	T
2	0.011	16	**C**	**delCA**	T	**A**	**G**	**A**	**C**	**insA**	**T**	C	C	G	G	C	C	G	C	G	C	G	**C**	**G**	T

Variants

1 IVS2+34 G > C, 2 IVS4+46 delCA, 3 IVS5+23 T > A, 4 IVS5-18 G > A, 5 IVS6-53 A- > G, 6 IVS8-52 T- > A, 7 ex10 824 C- > T, Arg218Trp*, 8 IVS12-37 insA, 9 IVS14+32 G > T, 10 ex15 2553 C > G, Ala794Gly*, 11 ex15 2656 C > T, Gly828Gly, 12 ex15 3087 G > A, Arg972Gln*, 13 ex15 4184 G > A, Glu1338Lys*, 14 ex15 4583 C > T, Arg1471Trp*, 15 IVS18+15 C > G, 16 ex17 4957 G > A, Lys1595Lys, 17 ex 18 5165 C > T, Arg1665Cys*, 18 ex 20 5507 G > A, Asp1779Asn, 19 ex 21 5791 C > T, Ala1873Ala, 20 ex 25 6255 G > A, Arg2027Gln, 21 3' UTR 6517 A > C, 22 3' UTR 6519 C > G, 23 3' UTR 6580 T > C

*variant that was further screened in familial breast cancer cases

## Discussion

Kammerer et al. utilized a genome-wide association analysis to identify breast cancer susceptibility regions and identified, among other regions, a high-linkage disequilibrium region on chromosome 11q13 [1]. This region contains several genes and *NuMA *was chosen as a most likely candidate for breast cancer susceptibility gene, and variant Ala794Gly was hypothesized to be functionally impaired and suggested to be associated with breast cancer risk [1].

Our thorough screening of *NuMA *gene in breast cancer cases resulted in identification of several variants of which eight were missense changes and the rest were synonymous variants or not located in coding regions. Missense variants that warranted further screening after bioinformatic analyses were present in the breast cancer cases in similar frequencies as in controls. Variant Arg1471Trp was not detected in controls, however, but being present in only 0.6% of the cases would have only marginal effect on breast cancer even if having any effect on breast cancer risk for the carriers. These variants represented also unique haplotypes, not suggesting presence of other risk alleles in linkage disequilibrium with these variants either, although due to low power, very low risk alleles may go unrecognized.

We also specifically evaluated the variant *NuMA *Ala794Gly for breast cancer risk in an extensive patient series (884 unselected cases, 910 familial cases and 906 population controls) as compared to those used by Kammerer et al, (2005) [1]. For this analysis, our material has 98% power to detect a difference in frequency of that magnitude.

Variant Ala794Gly was detected in almost equal frequencies in all our study series as well as in population controls, which is consistent with the results from a large breast cancer patient and control series recently studied by the Breast Cancer Association Consortium [24] and does not support the previously proposed association with breast cancer risk. However, as the variant Ala794Gly was initially found here to be present in two distinct haplotypes defined by an intronic variant it is possible that differences in the relative haplotype frequencies could have masked any associated risk. Further evaluation of these two haplotypes, however, did not support this possibility. None of the identified *NuMA *variants was associated with breast cancer risk in our study. Furthermore, Ala794Gly variant was not significantly associated with any of the tumor characteristics.

## Conclusion

In conclusion, our results do not support the role of *NuMA *variants as breast cancer risk alleles.

## Abbreviations

NuMA: Nuclear Mitotic Apparatus protein; SNP: Single nucleotide polymorphism; APL: Acute promyelocytic leukemia; RARα: Retinoic acid receptor α; PML: promyelocytic leukemia protein; GAS41: Glioma-amplified sequence 41; SSCP: Single strand conformation polymorphism; CSGE: Conformation sensitive gel electrophoresis; RFLP: Restriction fragment length polymorphism; UTP: Untranslated region.

## Competing interests

The authors declare that they have no competing interests.

## Authors' contributions

OK did the statistical analyses and drafted the manuscript and did some of the molecular genetic studies. MR and AT were responsible for the majority of molecular genetic studies involving screening of *NuMA*. KA and CB collected the patient samples and clinical data in the study. HN participated in the study design and helped in drafting the manuscript.

## Pre-publication history

The pre-publication history for this paper can be accessed here:


